# Comparing Salivary Caffeine Kinetics of ^13^C and ^12^C Caffeine for Gastric Emptying of 50 mL Water

**DOI:** 10.3390/pharmaceutics15020328

**Published:** 2023-01-18

**Authors:** Michael Grimm, Adrian Rump, Lisa Meilicke, Maximilian Feldmüller, Rebecca Keßler, Eberhard Scheuch, Mladen Vassilev Tzvetkov, Werner Weitschies

**Affiliations:** 1 Department of Biopharmaceutics and Pharmaceutical Technology, University of Greifswald, 17489 Greifswald, Germany; 2 Department of Clinical Pharmacology, Bayer AG Pharmaceuticals, 13353 Berlin, Germany; 3 Department of Diagnostic Radiology and Neuroradiology, University Hospital Greifswald, 17475 Greifswald, Germany; 4 Department of Clinical Pharmacology, University Hospital Greifswald, 17487 Greifswald, Germany

**Keywords:** gastric emptying, magnetic resonance imaging (MRI), caffeine, saliva tracers, UNGAP, stable isotopes

## Abstract

Gastric water emptying as a critical parameter for oral drug absorption can be investigated by several imaging techniques or by the interpretation of pharmacokinetics of appropriate substances. Recently introduced salivary caffeine kinetics is a valuable tool, but the required caffeine abstinence limits its applicability. To avoid the caffeine abstinence, stable isotope-labeled caffeine might be used, but the representability and transferability of kinetics for evaluation of gastric emptying must be demonstrated. Thus, salivary caffeine pharmacokinetics were compared for naturally occurring ^12^C-caffeine and ^13^C_3_-caffeine after the administration of water under fasting conditions in six healthy young subjects. For this purpose, an ice capsule containing the two caffeine species was administered with 50 mL tap water. Gastric water emptying was simultaneously quantified using magnetic resonance imaging (MRI). Gastric emptying of 50 mL of water could be successfully evaluated. The salivary caffeine kinetics of ^13^C_3_- and ^12^C-caffeine were nearly congruent and showed good linear correlations in all subjects, with a mean correlation coefficient of 0.96 in pooled data. Thus, the substitution of natural ^12^C caffeine with stable isotope-labeled ^13^C_3_-caffeine offers the opportunity for broader application of the salivary caffeine gastric emptying technique and increases the robustness of the method against environmental contamination with caffeine.

## 1. Introduction

Gastric emptying is a prerequisite for the absorption of most drugs and is affected by various factors such as the administered fluid volumes, fluid types, meal intake, caloric load and more. It can be investigated using several techniques including breath tests or imaging techniques such as scintigraphy, 3D-ultrasonography or magnetic resonance imaging (MRI) [1]. Despite their advantages, the use of imaging techniques is often costly and time-consuming. Moreover, gastric volume measurements can be affected by secretion and might not sufficiently represent transfer from the stomach to the small intestine in the case of lower gastric volumes, as recently shown [2].

By the use of sufficiently soluble and rapidly absorbable substances, pharmacokinetics can also be used to evaluate gastric emptying. For the quantification of mass transfer to the small intestine, pharmacokinetics-based techniques might even be the better predictor. Mainly paracetamol (acetaminophen) [3,4,5,6,7] and caffeine [8,9,10] are used as probe drugs for the evaluation of gastric emptying. These probe drugs can be used as solutions to represent gastric emptying of water or other fluids, or they can be administered as gastroresistent pellets, although their homogeneous distribution in media is questionable and the lag time until coating solubilization needs to be considered, possibly hindering the evaluation of fast emptying processes. To avoid blood sampling for pharmacokinetic evaluation, paracetamol can also be detected in saliva [5,11]. This also applies to caffeine; gastric emptying related to the correlation of caffeine in saliva and blood plasma can be evaluated noninvasively by salivary sampling [10,12,13]. In this regard, caffeine might be favorable due to its higher solubilization rate and lower doses needed. Furthermore, the salivary sampling technique utilizing caffeine can also be applied to investigate the onset of dosage form disintegration, due to its rapid dissolution [14,15,16,17].

Nonetheless, the broader application is hindered by the necessary but inconvenient caffeine abstinence of the subjects. To overcome this problem, stable isotope-labeled caffeine might be used instead of the naturally occurring ^12^C-caffeine. Stable isotope-labeled molecules have been used for many years in pharmacokinetic studies [18,19] avoiding radiation exposure. Deuterated (^2^D) substances are most often used, but ^15^N or ^13^C labeled substances are also common. For caffeine, different isotope-labeled molecules with different signals in mass spectroscopy analytics are available. Frequently, the methylene groups of 1,3,7 trimethylxanthine (caffeine) are labeled with the stable carbon isotope ^13^C, so that one to three labeled caffeine identities can be easily used. These molecules are theoretically not different in their chemical properties and just differ in molecular mass. Accordingly, ^13^C_3_-caffeine has been used to avoid caffeine abstinence in studies evaluating dosage form disintegration, where the appearance of this unnatural species in saliva turned out to be a good indicator of capsule rupture [14,16]. Nonetheless, it remains to be demonstrated that the pharmacokinetics of stable isotope-labeled substances are comparable to those of the natural compound consisting of the most frequently occurring isotopes.

It was the aim of the present work to prove the usefulness and comparability of stable isotope labeling for the salivary caffeine technique. Thus, ^13^C_3_-caffeine was used in parallel with ^12^C-caffeine for the evaluation of gastric emptying of 50 mL of water. Gastric emptying was further evaluated by the established MRI technique to evaluate the effect of an intermediate volume on gastric emptying and compare it to recent data on the gastric emptying of 20 mL and 240 mL of water.

## 2. Materials and Methods

### 2.1. Study Materials

Conventional ^12^C-caffeine was purchased from Fagron GmbH & Co. KG (Barsbüttel, Germany). Isotope-labeled ^13^C_3_-caffeine was purchased from Sigma-Aldrich (Steinheim, Germany). In addition to the certificate of analysis for isotope-labeled caffeine, in-house measurements of NIR, LC-MS and NMR assured the chemical and isotopic purity of the substance. Saccharin sodium was obtained from Caelo (Hilden, Germany). Formic acid and ammonium acetate were obtained from Merck KaA GmbH, (Darmstadt, Germany). Acetonitrile, methanol and water used for LC-MS were purchased from VWR International (Fontenay sous Bois, France). Silicon for the preparation of casting molds for ice capsules was of food quality and obtained from Altropol GmbH (Stockelsdorf, Germany). The water administered to the subjects was tap water supplied by a local water company (Greifswald, Germany).

### 2.2. Study Design

To evaluate the utilization of stable isotope-labeled caffeine for the evaluation of gastric water emptying in humans, the previously published study comparing the gastric emptying of 20 mL and 240 mL was amended to a 3-way open label crossover study combining pharmacokinetics and MRI after intake of 50 mL of water [2]. Ethical approval was obtained from the Ethics Committee of the University Hospital Greifswald (BB 071/17a). The study was conducted according to German MPG §23b, Good Clinical Practice Guidance, the Professional Code for Physicians in Germany and the Declaration of Helsinki.

Of the original 8 subjects, 6 healthy volunteers (4 males, 2 females) with a mean age of 24.7 ± 1.1 years and a mean BMI of 24.3 ± 2.1 kg/m^2^ were included again. The other 2 subjects were no longer contactable. The inclusion criteria remained adapted to FDA and EMA guidelines for bioavailability and bioequivalence studies. All subjects were nonsmokers. None of the subjects had a history of gastrointestinal disorders or gastrointestinal surgery, or a history of alcohol or drug abuse. During the study procedures, no subject took any medication known to affect GI physiology. Insurance was obtained for commuting accidents and any harm from study procedures.

### 2.3. Study Protocol

Imaging was conducted at the Department of Radiology of the University Medicine Greifswald. The subjects self-reliantly arrived there in the morning after at least 10 h of fasting overnight. To ensure sufficiently low basal ^12^C-caffeine concentrations in saliva, the subjects had to abstain from caffeine and food containing caffeine such as tea, coffee, chocolate and soft drinks for at least 72 h. Furthermore, the subjects had to abstain from alcohol 48 h before the study procedures. On the study day, the subjects received 50 mL of water together with an ice capsule containing 35 mg of ^12^C-caffeine and 35 mg of ^13^C_3_-caffeine. The administration was carried out in an upright position. Immediately after the administration, the subjects flushed out their mouth with water thoroughly to avoid contamination of the oral cavity from caffeine residues potentially attached to the ice capsule.

Gastric volumes were evaluated using magnetic resonance imaging (MRI). The concentrations of both caffeine species were determined from saliva. The intake of the ice capsule with water was defined as time point t = 0 min. Abdominal MR imaging was performed before intake of water and the ice capsule (t = −5 min) and at time points 2 min, 4 min, 6 min, 8 min, 10 min, 12 min, 14 min, 16 min, 18 min, 20 min, 25 min, 30 min, 35 min, 40 min, 50 min and 60 min. The saliva probes were given self-reliantly 1 min after the respective MRI sequence, and additionally at time points 90 min, 120 min, 150 min, 180 min, 210 min and 240 min. After each study day, the racks with the probes were frozen at −80 °C and stored until analysis.

### 2.4. Preparation and Labeling of Ice Capsules

As previously described, ice capsules can be used to label the administered water with caffeine, avoiding contamination of the oral cavity. This way, gastric emptying can be evaluated by salivary caffeine kinetics as described by Sager et al. [10]. Preparation of ice capsules was performed as described previously [10]. In order to incorporate both ^12^C-caffeine and ^13^C_3_-caffeine, the filling of the frozen capsule shell consisted of 0.5 mL of a pre-cooled solution with 35 mg of naturally occurring caffeine (^12^C-caffeine), 35 mg of stable isotope-labeled ^13^C_3_-caffeine as well as 250 mg of saccharine sodium, to increase the solubility of caffeine. After complete freezing at −80 °C, the capsules were stored at −10 °C until administration. The molten ice capsules had a volume of 1.45 mL, which is regarded as negligible in terms of gastric volume kinetics.

### 2.5. Analysis of Salivary Caffeine Concentrations

Frozen saliva samples were thawed for 1 h. Afterwards, the micro tubes were centrifuged at 13,000 rpm for 15 min (Biofuge pico, Heraeus, Germany). For protein precipitation, 400 µL of a solution composed of acetonitrile with 6% formic acid was mixed with 200 µL of the supernatant in a 1.5 mL micro tube (Sarstedt, Nümbrecht, Germany). The mixture was vortexed at maximum speed for 1 min (VORTEX 2, IKA^®^-Werke GmbH & Co. KG, Staufen, Germany). Subsequently, the mixture was centrifuged at 13,000 rpm for 15 min. A volume of 150 µL of the supernatant was placed in 300 µL vials (ND9, PP braun, 32 × 11.6 mm, neoLab, Heidelberg, Germany) and diluted with 150 µL LC-MS-grade water containing 4% formic acid. Subsequently, the probes were vortexed at maximum speed for 1 min again. Afterwards, the samples were used for analysis. Quantification of salivary caffeine was performed as described previously [10] using an Agilent 1100 series HPLC system (Agilent Technologies, Waldbronn, Germany) coupled to the triple quadrupole mass spectrometer API4000 QTRAP with the electrospray ionization source Turbo V™. The whole system was controlled by Analyst 1.6 software (AB Sciex, Darmstadt, Germany).

An isocratic elution with ammonium acetate buffer (5 mM; pH 3.8) (A)/methanol (B) (50/50) as the mobile phase with a flow rate of 250 μL/min was used. The temperature of XTerra^®^MS reverse phase C18 column (3.5 µm, 2.1 × 100 mm; Waters, Dublin, Ireland) was set to 40 °C and the injection volume was 20 µL. A 0.5 µm pre-filter (PEEK, Supelco, Taufkirchen, Germany) was used to avoid particulate contamination. Ionization was performed using an ESI interface (Turbo V™ ionization source) in positive ionization mode. Gas parameters were as follows: temperature, 550 °C; gas 1, 60 psi; gas 2, 60 psi (both nitrogen); voltage, 4000 V; collision-activated dissociation (CAD), 12 (arbitrary unit). The chromatograms were evaluated by Analyst 1.6 software using an internal standard method and peak–area ratios for calculation (quadratic regression, 1/x weighting). The whole analytical method was validated concerning linearity, precision, accuracy, selectivity and freeze/thaw stability according to the FDA guidance “Bioanalytical Method Validation” (Issue May 2001). The lower limit of quantification was 5 ng/mL for caffeine in saliva.

### 2.6. Magnetic Resonance Imaging

A 1.5 Tesla MRI scanner (Siemens MAGNETOM Aera) at the Department of Diagnostic Radiology and Neuroradiology of University Medicine Greifswald was used for imaging procedures. MRI measurements were carried out in the supine position in head forward configuration. For signal detection, a 6-element phase array abdominal coil on the subjects’ abdomen and four spine coils inside the MRI desk were used as receiver coils. Acquisition was performed with software syngo MR E11 implemented in the console terminal of the tomograph (Siemens Healthcare, Erlangen, Germany).

For the evaluation of gastric volumes, strongly T2 weighted HASTE sequences were used. The sequences had a TR of 1000 ms, a TE of 198 ms, a slice thickness of 5 mm, an interslice gap of 1 mm, voxel size of 12.2 mm^3^ and a variable flip angle of 130–180° depending on individual SAR related to weight and room temperature. Due to the variable number of slices according to the different weights of the subjects, the acquisition time varied between 25 and 35 s. During that time, the subjects had to hold their breath to reduce motion artifacts.

### 2.7. Image Analysis

The images were further analyzed using Horos v2.2.0 software (The Horos Project). The liquid filling the stomach could be easily distinguished from the surrounding structures, due to the pronounced T2 weighted contrast. The gastric filling was manually marked as a region of interest (ROI) in every single image slice. Using the marked surface area of ROI and known slice thickness and interslice gap, the volumes could be calculated using an integrated software tool of Horos.

### 2.8. Saliva Sampling, Sample Preparation and Analysis

The saliva samples were drawn by the subjects self-reliantly into 2 mL reaction tubes. After each study day, the probes were stored in a freezer (−80 °C) until analysis. Sampling, preparation and analysis of salivary probes were performed as previously described [2,10]. The entire analytical method was validated according to the FDA guidance “Bioanalytical Method Validation” (Issue May 2001), concerning linearity, precision, accuracy, selectivity and freeze/thaw stability. For both caffeine species, the lower limit of quantification was 5 ng/mL in saliva.

### 2.9. Normalization of Caffeine Concentration and MRI Volume Data

For comparability, the obtained ^12^C-caffeine salivary concentrations and gastric volume data (MRI) were normalized, as described before [10]. For this aim, caffeine concentrations were divided by c_max_. A relative caffeine concentration of 100% therefore represents c_max_. For an oral caffeine solution in fasted state, it has already been shown that c_max_ corresponds well to the total gastric emptying, which is why this assumption was also made here. The normalization of gastric content volumes from MRI evaluations was performed as described by Grimm et al. [20]. Individually acquired resting volume before administration plus ingested volume (50 mL in this study) was set to 0% emptying. Therefore, 100% emptied volume represents the complete emptying of fluid that was present in the stomach right after ingestion of the ice capsule.

### 2.10. Statistical Analysis

For graphical illustrations and linear correlations of data, OriginPro 8.5.1G (OriginLab Corporation, Northampton, MA, USA) was used. The adjusted coefficient of determination (R²) of linear regression was employed for the evaluation of the linear correlations. Statistical calculations were performed with GraphPad Prism 5 (GraphPad Software Inc., Boston, MA, USA). t_max_, c_max_ as well as residual fasted gastric content volume (fGCV) for treatment comparability were obtained from raw data. Relative gastric emptying at 30 min (rel.GE_30min_) was obtained from normalized relative volume data. Area under the curve (AUC) and area under the volume curve (AUVC) were calculated from raw relative data using the trapezoidal rule in Microsoft^®^ Excel^®^ 2013 (Microsoft Corporation, Redmond, WA, USA). Before performing a statistical comparison, the data were tested for normality using the Kolmogorov–Smirnov and Shapiro–Wilk tests. Since data were not normally distributed, we applied the non-parametric Wilcoxon signed rank test for the comparison of both caffeine species or the Friedman test with Dunn’s post-test for the comparison of volume kinetics of 50 mL with previously acquired 20 mL and 240 mL data.

## 3. Results

The study was conducted successfully without any adverse events. All six recruited volunteers completed the additional study treatment. Swallowing of the ice capsule was reported to be difficult by some subjects again, but no salivary caffeine contaminations were observed due to the ice capsule having already melted in the mouth. All MR images were evaluable and gastric content volumes could be analyzed. The salivary probes of subject 001 at time point 7 min were not evaluable and were therefore excluded. All other calculations (e.g., AUC) were adapted accordingly.

### 3.1. Correlation of Gastric Emptying and Pharmacokinetics

**Figure 1:**
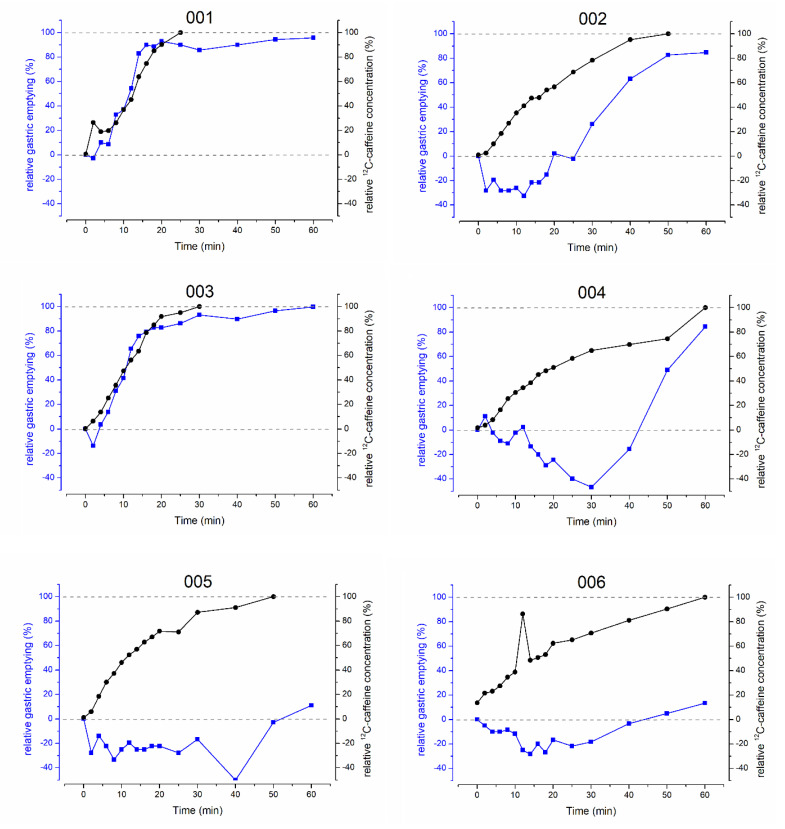
Individual relative gastric emptying (blue) and salivary ^12^C-caffeine concentrations (black) before and after intake of 35 mg caffeine with 50 mL of water.

It was one aim of the study to quantify the gastric emptying of 50 mL and to compare it with the gastric emptying of 20 mL and 240 mL. Thus, the main pharmacokinetic parameters of ^12^C-caffeine representing gastric emptying as well as the area under the gastric volume curve (AUVC) and rel.GE_30min_ were compared. For the evaluation of the comparability of the starting conditions, fGCV was added. Statistical comparisons are shown in Table 1. Individual comparisons of relative gastric emptying and relative salivary caffeine concentrations are shown in Figure 1.

### 3.2. Comparison of Caffeine Isotope Pharmacokinetics

**Figure 2:**
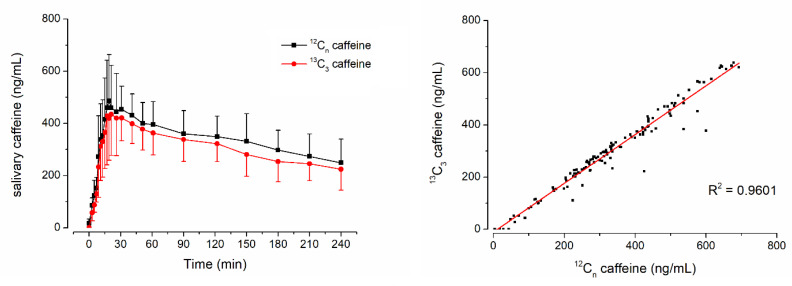
Mean ± SD salivary concentrations of ^12^C-caffeine, ^13^C_3_-caffeine and the individual linear correlation of the respective concentrations from six volunteers after administration of an ice capsule containing 35 mg ^12^C-caffeine and 35 mg ^13^C_3_-caffeine together with 50 mL tap water.

**Figure 3:**
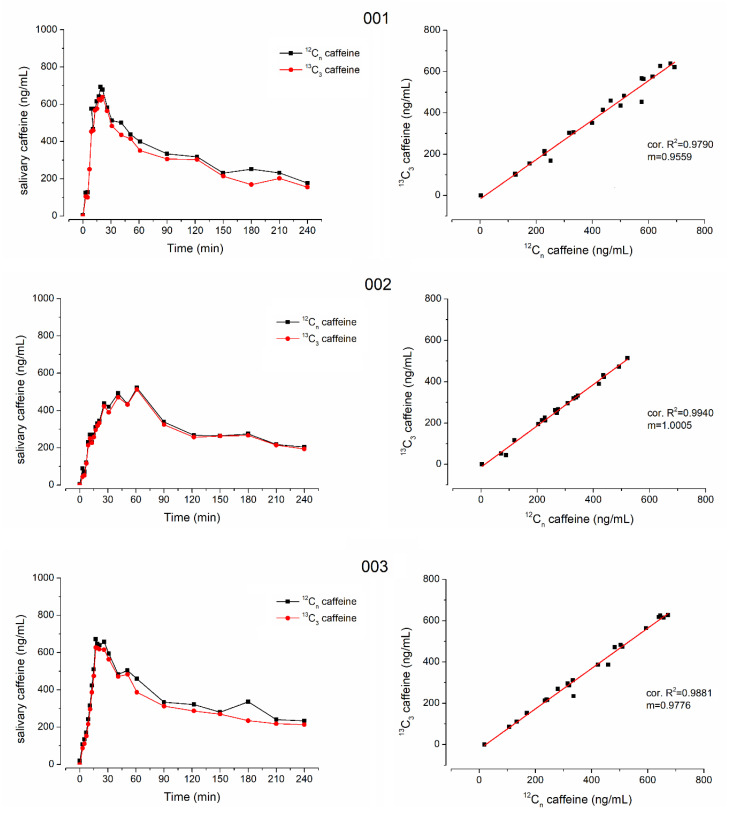
Individual salivary caffeine concentrations of ^12^C-caffeine (black) and ^13^C_3_-caffeine (red) on the left side and their linear correlation on the right side from subjects 001, 002 and 003.

**Figure 4:**
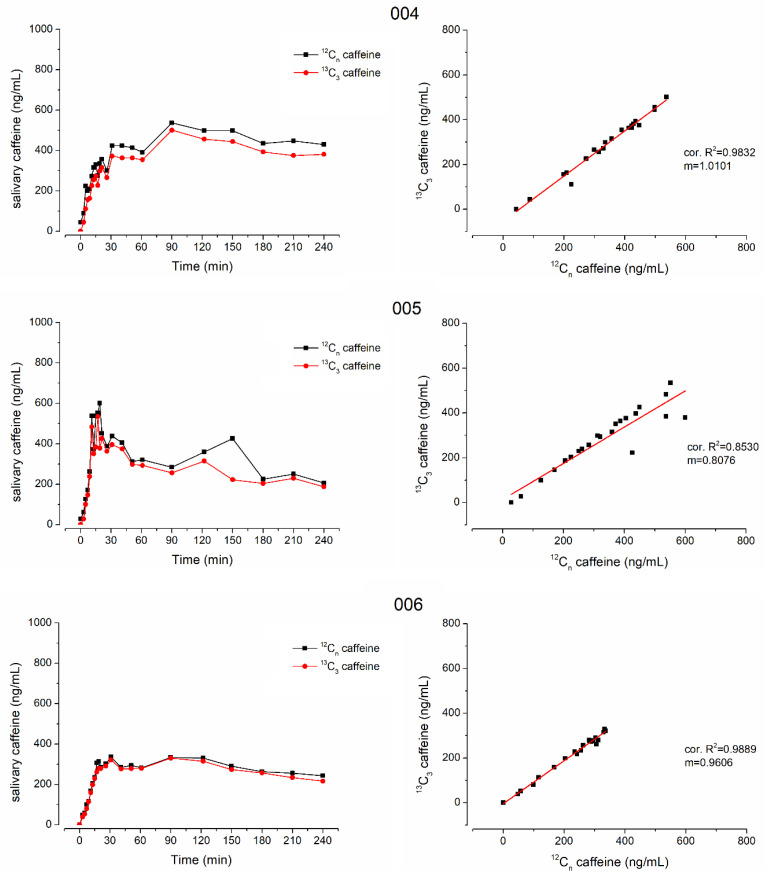
Individual salivary caffeine concentrations of ^12^C-caffeine (black) and ^13^C_3_-caffeine (red) on the left side and their linear correlation on the right side from subjects 004, 005 and 006.

Another question of this study was whether stable isotope-labeled ^13^C_3_-caffeine could be used for salivary sampling studies to determine gastric emptying. To evaluate this question, the ice capsule administered with 50 mL of water was additionally labeled with 35 mg of ^13^C_3_-caffeine. In Figure 2, the mean pharmacokinetic profiles of ^12^C-caffeine and ^13^C_3_-caffeine as well as the pooled linear correlation of the respective concentrations are shown. Mean pharmacokinetic parameters and the respective statistical comparison of ^12^C-caffeine and ^13^C_3_-caffeine are given in Table 2. The respective individual curves of each subject are displayed in Figure 3 and Figure 4.

In all subjects who received isotope-labeled ^13^C_3_-caffeine, the concentrations of natural and isotope-labeled caffeine correlated well with only few exceptions due to data points that were mainly also outliers in the individual profiles, e.g., time points 40 min of subject 005 and 12 min of subject 006. Moreover, in general, the concentrations were not identical and the isotope-labeled ^13^C_3_-caffeine had a significantly smaller c_max_ and AUC (*p* < 0.05 Wilcoxon signed rank test). Nonetheless, t_max_ did not differ significantly, which is of main importance together with good linear correlation. The minor difference in mean t_max_ is attributed to the double peak shape of caffeine pharmacokinetics in subject 6. For ^13^C_3_-caffeine, the second peak is slightly higher than the first one, leading to an unrepresentative increase in mean t_max_.

## 4. Discussion

Residual fasted gastric content volume (fGCV) and gastric emptying were well in line with the literature [21], and gastric emptying of 50 mL of water was as expected from a recent publication [2]. The relative gastric volume decrease of 50 mL was slower than after 240 mL but faster than after 20 mL of water, even though no statistical significance was achieved for rel.GE_30min_ or AUVC. This might be attributed to the high variability of the volume kinetics and the limited sample size for this exploratory study. Caffeine absorption was comparable irrespective of ingested volume, confirming a comparable transfer from the stomach to the small intestine. As discussed in a recent publication [2], differences in volume kinetics are probably mainly attributed to the pronounced influence of secretion on volume measurements of lower volumes, whereas the real transfer of content from the stomach to the small intestine is more robustly estimated by caffeine kinetics. Secretions could also lead to an apparent time lag in gastric emptying, since volume did not decrease or even increased during the evaluated time. Unstimulated gastric secretion rates in a fasted state are reported to be around 1 mL/min [22,23] or 0.9 ± 0.2 mL/min [24]. However, Yamashita et al. calculated mean fasted secretion rates of up to 7.16 mL/min or 2.78 mL/min, respectively, averaged over a day [25]. Not only emptying rates but also secretion rates are dependent on MMC, which probably also contributes to the observed higher variability after intake of lower volumes [26]. These secretion rates can peak to up to 400 µmol/min of HCL in phase II of MMC [26]. Assuming a concentration of 0.1 mol/L, that would correspond to 4 mL/min only of HCL solution, excluding gastric bicarbonate or salivary secretion volumes. Since secretion is also dependent on mental factors such as hunger or appetite, even speaking about an upcoming meal after completion of the study could contribute to an increase in gastric volume. Nonetheless, data on unstimulated gastric secretions need to be considered with caution, as all direct or indirect techniques for the evaluation of gastric secretion have specific pitfalls, which are not extensively discussed within this work. However, the data show that secretion rates can exceed emptying rates in a fasted state, which becomes even more likely the lower the ingested fluid volume is.

These factors most likely led to negative apparent emptying rates in some subjects within a limited time frame. Nonetheless, caffeine kinetics showed that absorption and thus gastric emptying occurred at the same time. In this regard, the applicability of MRI for volume measurements of gastric contents, e.g., for the estimation of dissolution media, is still robust, but the applicability of MRI for the estimation of gastric emptying of small volumes seems rather limited [2]. As expected, the variability of gastric emptying after intake of 50 mL was higher than after 240 mL, most likely since secretory activity of the stomach as well as the current phase of MMC during gastric emptying may contribute to a higher extent in the case of the lower volume [2].

The higher relevance of MMC on gastric emptying of lower volumes was previously highlighted by Oberle et al. using a non-absorbable marker technique, showing that apparent gastric emptying of 50 mL is more dependent on MMC than the emptying of 200 mL [27]. Moreover, the gastric emptying after 200 mL appeared to be faster than that of 50 mL, as is the case with our volumetric data. The higher the volume is, the less dependent it might be on active motility, since hydrostatic pressure and gastric wall tension as driving forces are probably increased. It needs to be considered that these data from another method are sensitive to secretory activity as well, due to small intestinal concentrations measured but not considering the volumes. A lower concentration of duodenum could result from slower gastric emptying, as interpreted by the authors, or it could be related to the same gastric emptying but a relatively higher amount of secretion related to intake volume. Thus, the results form Oberle et al. are in line with our observations. Another study using ultrasonography and pharmacokinetics of paracetamol (acetaminophen) revealed the same results. The gastric volume decrease was higher after the intake of 300 mL compared to 50 mL, but paracetamol kinetics were the same, indicating the same transfer rates from the stomach to the small intestine. Even though this study was performed with pregnant women, the results of this combined imaging plus pharmacokinetic study are comparable to our imaging plus pharmacokinetic observations [28].

As is common in MRI studies, the subjects were in the supine position during our experiment. Recent work highlighted the effect of body position on gastric emptying, and the effect of different volumes on gastric emptying might be different in the upright position [29,30]. Nonetheless, the two previously mentioned studies by Oberle et al. and Wong et al. were carried out mainly in the upright position, and comparable findings on the gastric emptying rates of different volumes were observed. Thus, the effect of body position might change gastric emptying rates in general but does not change the relative effect of different volumes on gastric emptying. Apparent gastric emptying in terms of volume change of non-caloric fluids is probably slower the lower the intake volume is, but gastric emptying in terms of transfer to the small intestine seems to be comparable irrespective of the intake volume.

Under clinical standard conditions with 240 mL of water, it has already been shown that gastric emptying correlated well with ^12^C-caffeine absorption [10]. Apart from the comparison of gastric emptying of different volumes, it was the main issue of the present work to establish an isotope-labeled alternative to naturally occurring caffeine. The comparison of ^12^C-caffeine and stable isotope-labeled ^13^C_3_-caffeine concentration in saliva revealed a good linear relation. Nonetheless, the c_max_ and AUC of ^13^C_3_-caffeine was significantly smaller, although 35 mg of both caffeine species was administered. This might be related to the manual preparation of the ice capsules and the pipetting of supersaturated caffeine solutions under low temperature, leading to differences in dosing, but this did not affect linear correlation. Moreover, for studies on gastric emptying, the absorption part of the curve and t_max_ are of main importance, which did not differ relevantly.

The reason for lower exposure after intake of ^13^C_3_-caffeine could also be attributed to the isotopic purity of the labeled substance. However, unlabeled caffeine or partially labeled ^13^C_2_-caffeine and ^13^C_1_-caffeine were not observed in the purity analysis of the ^13^C_3_-caffeine used. During the experiments, small amounts of ^13^C_2_-caffeine and ^13^C_1_-caffeine were detected in salivary probes with increasing time, but the responsible mechanisms remain unclear. It could be that concentrations at the beginning were too low to be quantified, or that ^13^C_2_-caffeine and ^13^C_1_-caffeine are formed during the experiment in vivo, e.g., by methylene group transfer. Irrespective of the reason for the presence of ^13^C_2_-caffeine and ^13^C_1_-caffeine, their occurrence could have accounted for a decrease in ^13^C_3_-caffeine compared to ^12^C-caffeine. Lower absorption of labeled caffeine or slightly faster metabolism could also lead to lower exposure of ^13^C_3_-caffeine, but this seems unlikely.

Overall, a good linear relationship between ^12^C-caffeine and ^13^C_3_-caffeine concentrations was confirmed, indicating that salivary ^13^C_3_-caffeine pharmacokinetics can be used for the determination of gastric emptying. This is in line with the common use of isotope-labeled drug molecules in early pharmacokinetic studies [18,19]. Nonetheless, in a recently published study, it was observed that entirely deuterated D_9_-caffeine yielded a 4-fold increase in AUC compared to natural caffeine, indicating that isotope labeling might sometimes indeed alter pharmacokinetics in a relevant way [31]. This was not the case for ^13^C labeling.

**Figure 5:**
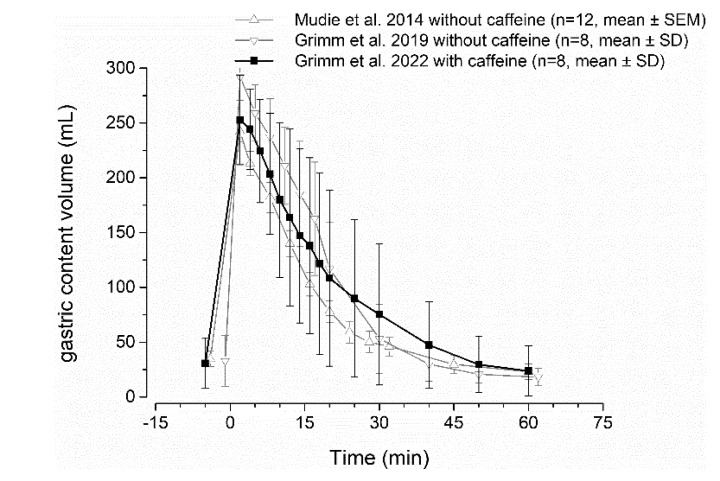
Gastric volume curves after intake of 240 mL of water with 35 mg caffeine (black) and without caffeine (grey) from literature [2,36,37].

For the broader application of the salivary sampling technique for biopharmaceutical questions, it needs to be confirmed that caffeine has no influence on gastric emptying and other relevant gastrointestinal parameters. An accelerating effect on gastric emptying is reported for coffee [32,33]. In contrast to that, a decelerating effect of coffee on gastric emptying by prolonging adaptive relaxation of the stomach has also been observed [34]. Thus, the effect of coffee on gastric emptying remains unclear. Others also reported no effect of coffee on gastric emptying of liquid meals [35]. The comparability of these studies with our caffeine method is limited, as caloric liquids or meals were most often used, which might not be representative of non-caloric fluids such as water in this study. Moreover, caffeine is most probably not the substance responsible for the effects of coffee on gastrointestinal function, as regular coffee and decaffeinated coffee have an identical effect on gastrointestinal function [34]. Even if caffeine itself would show a relevant effect on gastrointestinal physiology, the amount of caffeine used for fluid labeling is very small. To further prove no relevant influence of salivary caffeine method on gastric emptying, we performed a comparison of literature data. Comparing data for emptying 240 mL of water with and without caffeine administered with an ice capsule, no relevant effect of caffeine addition can be observed, as shown in Figure 5. Thus, it can be concluded that the method does not affect gastric emptying.

Besides its effect on gastric emptying, coffee also stimulates gastrin release and gastric acid secretion. This is most likely related to other compounds in coffee rather than caffeine. Pure caffeine was shown not to affect gastrin release in a relevant way [34]. Thus, an effect of the method on gastric pH is also unlikely. Since no effects on gastric physiology are to be expected, the necessity of caffeine fasting can be avoided by the use of stable isotope-labeled caffeine.

## 5. Conclusions

Stable isotope labeling of caffeine with ^13^C as a salivary sampling technique will ease further investigation of gastric emptying and disintegration of solid oral dosage forms without the need to abstain from food and beverages containing caffeine. If gastrointestinal localization of dosage form and specific gastrointestinal fluid amounts are not of interest, even additional imaging can be waived.

## Figures and Tables

**Table 1:** Mean ± SD pharmacokinetic parameter of ^12^C-caffeine representing gastric emptying of 20 mL, 50 mL or 240 mL as well as rel.GE_30min_ and gastric AUVC (n = 6 each), with asterisk indicating significant difference according to Friedman’s ANOVA with Dunn’s post-test (n.s. = not significant).

		20 mL	50 mL	240 mL	Statistics
t_max_	(min)	46.8 ± 15.0	39.5 ± 29.8	42.7 ± 9.8	n.s.
c_max_	(ng/mL)	434 ± 84	560 ± 130	432 ± 106	n.s.
AUC_0-tlast_	(min∙μg/mL)	66.2 ± 11.8	80.7 ± 13.2	68.5 ± 20.4	n.s.
AUVC_0-tlast_	(min %)	4512 ± 2545	3181 ± 2360	2237 ± 987	n.s.
rel.GE_30min_	(%)	26 ± 47	57 ± 40	73 ± 20	n.s.
fGCV	(mL)	24 ± 14	15 ± 15	31 ± 21	n.s.

**Table 2:** Mean ± SD pharmacokinetic parameter of ^12^C-caffeine and ^13^C_3_-caffeine (n = 6), with asterisk indicating significant difference according to Wilcoxon signed rank test (n.s. = not significant).

		^12^C-Caffeine	^13^C_3_-Caffeine	Statistics
t_max_	(min)	39.5 ± 29.8	49.3 ± 35.6	n.s. *p* = 0.59
c_max_	(ng/mL)	560.0 ± 129.6	524.0 ± 111.6	*p* = 0.03
AUC	(min∙μg/mL)	80.7 ± 13.2	72.9 ± 11.0	*p* = 0.03

## Data Availability

The data can be shared up on request.
